# Real-world effectiveness of abatacept for rheumatoid arthritis treatment in European and Canadian populations: a 6-month interim analysis of the 2-year, observational, prospective ACTION study

**DOI:** 10.1186/1471-2474-15-14

**Published:** 2014-01-11

**Authors:** Hubert G Nüßlein, Rieke Alten, Mauro Galeazzi, Hanns-Martin Lorenz, Dimitrios Boumpas, Michael T Nurmohamed, William G Bensen, Gerd R Burmester, Hans-Hartmut Peter, Franz Rainer, Karel Pavelka, Melanie Chartier, Coralie Poncet, Christiane Rauch, Manuela Le Bars

**Affiliations:** 1 University of Erlangen-Nuremberg, Nuremberg, Germany; 2 Schlosspark-Klinik, University Medicine, Berlin, Germany; 3 University of Siena, Siena, Italy; 4 University Hospital Heidelberg, Heidelberg, Germany; 5 University of Crete, Heraklion, Greece; 6 VU University Medical Center/Jan van Breemen Research Institute, Amsterdam, The Netherlands; 7 St. Joseph’s Hospital/McMaster University, Hamilton, Ontario, Canada; 8 Charité-Universitätsmedizin Berlin, Berlin, Germany; 9 University Medical Center Freiburg, Freiburg, Germany; 10 Hospital Barmherzige Brueder, Graz, Austria; 11 Institute of Rheumatology and Clinic of Rheumatology, Charles University, Prague, Czech Republic; 12 Chiltern International, Neuilly, France; 13 Docs International, Sèvres, France; 14 Bristol-Myers Squibb, Munich, Germany; 15 Bristol-Myers Squibb, Rueil-Malmaison, France

**Keywords:** Rheumatoid arthritis, Biological agents, Abatacept, Effectiveness, Safety, Registries

## Abstract

**Background:**

Discontinuation of rheumatoid arthritis (RA) treatment for lack or loss of initial response, tolerability issues, or development of antibodies against the therapeutic agent remains a challenge in clinical practice. Here we present a 6-month interim analysis of a 2-year prospective observational trial in Europe and Canada aiming to assess the real-world effectiveness, safety, and tolerability of intravenous abatacept for the treatment of moderate-to-severe RA.

**Methods:**

ACTION (AbataCepT In rOutiNe clinical practice) is a prospective, observational study assessing effectiveness, safety, and tolerability of abatacept in patients with RA enrolled in Europe and Canada between May 2008 and January 2011. The patient population was divided into two groups: biologic naïve (‘first-line’) patients and patients who had previously failed treatment with at least one biologic agent (‘second-line’). Retention rates were calculated using Kaplan–Meier curve estimates. Effectiveness was measured using European League Against Rheumatism (EULAR) response criteria, the 28-item Disease Activity Score, the Clinical Disease Activity Index (CDAI), and physical function, as assessed by the Health Assessment Questionnaire-Disability Index (HAQ-DI). Serious adverse events (SAEs) were reported for all enrolled patients.

**Results:**

Of 1138 consecutively enrolled patients, 1114 and 1079 patients were evaluable for retention and effectiveness, respectively. Overall, retention rates were 88.6% (95% confidence interval [CI]: 86.4, 90.4); 67.4% of patients achieved good/moderate EULAR response; 32.8% had a CDAI Low Disease Activity State (LDAS); and 44.7% a HAQ-DI response. Retention rates among first- and second-line patients were 93.0% (95% CI: 85.9, 96.6) and 88.1% (95% CI: 85.7, 90.0), respectively. The percentage of patients achieving CDAI LDAS was 40.0% (95% CI: 26.4, 53.6) for first- and 32.2% (95% CI: 28.4, 36.0) for second-line patients and the proportion achieving a HAQ-DI response was 60.3% (95% CI: 47.8, 72.9) versus 43.1% (95% CI: 39.0, 47.2), respectively. The incidence of SAEs was 4.7%.

**Conclusions:**

Evidence from this 6-month interim analysis suggests that abatacept offers an effective and well-tolerated treatment option for patients with RA, including those who have previously failed anti-tumor necrosis factor treatment. In addition, higher retention rates and effectiveness outcomes were observed when abatacept treatment was initiated earlier in the course of the disease.

## Background

The treatment of rheumatoid arthritis (RA) in routine clinical practice comprises both biologic and non-biologic disease-modifying anti-rheumatic drugs (DMARDs), including methotrexate (MTX) and tumor necrosis factor (TNF) blocking agents. Although these treatments are beneficial for many patients, some may not respond to treatment or may lose their initial response over time [1]. Indeed, anti-TNF therapy discontinuation rates in real-world practice are about 30%, based on cohort studies with median follow-up of 15–37 months [2,3]. In these studies, up to 50% of discontinuations were due to lack of efficacy and approximately 15–49% to safety issues [2,3]. Patients who experience lack of efficacy with one anti-TNF agent often have a poorer response to a second or third anti-TNF agent, reflecting loss of efficacy and increased resistance towards TNF-α blockade, which, in some cases is due to the development of anti-therapeutic antibodies [2-5]. This is demonstrated in several large cohort and retrospective studies by longer retention rates for first treatment courses versus subsequent courses (hazard ratio: 2.17; 95% confidence interval [CI]: 1.72, 2.58) and decreased median drug survival times for subsequent anti-TNF agents (37 months for first-line agent to 13 months for third-line agent) [3,5]. Recent data suggest that when treatment with an anti-TNF agent shows lack of efficacy, switching to a biologic agent with a different mechanism of action may be of benefit [6].

Randomized clinical trials (RCTs) of biologics have provided information on the efficacy, safety, and tolerability of treatment options in different patient populations in a clinical research setting. However, it has been reported that treatment response rates are lower in routine clinical practice compared with RCT evidence [7], possibly because of patient selection, the use of a washout period before inclusion, differences in dosing, comorbidities, and variable adherence to therapy [7]. Because patient populations in observational studies are not subject to the strict inclusion and exclusion criteria of RCTs, observational studies often include patients with different levels of disease activity and region-specific variations in treatment. Therefore, data from real-world observational studies often supplement the findings from RCTs [8].

Abatacept is a selective T-cell costimulation modulator [9]. Evidence from RCTs has demonstrated the efficacy, safety, and tolerability of abatacept for the treatment of moderate-to-severe RA in different patient populations [10-14]. Moreover, evidence from local registries in France [15], Denmark [16], and Sweden [17], as well as evidence from a small, single-site study of abatacept in routine clinical practice [18], support the findings from RCTs. However, the observations from these small, observational cohorts need to be validated in a larger cohort of patients treated in routine clinical practice over a longer period of time. The objective of this report is to present a 6-month interim analysis of the data from the ACTION (AbataCepT In rOutiNe clinical practice) study, a 2-year prospective, observational cohort study that enrolled patients with RA in Europe and Canada to evaluate patient retention and the effectiveness of treatment with abatacept in routine clinical practice.

## Methods

### Study design and patient population

ACTION was a non-interventional, international, multicenter, prospective, observational cohort study to evaluate patient retention and the effectiveness of intravenous abatacept treatment in patients with RA in Europe and Canada. Patients were enrolled prospectively between May 2008 and January 2011, either on, or within 3 months of, initiating treatment with abatacept according to the Summary of Product Characteristics (SmPC) in Europe and the Product Monograph in Canada. Patients already on treatment with the study drug (169/1114 [15.2%]) were included only if baseline data were available and could be collected retrospectively.

In all participating countries, abatacept was required to have market authorization and a reimbursement policy to ensure that eligible patients had access to the drug. No product was provided to physicians or patients by the study sponsor. This observational study did not interfere with a physician’s routine clinical practice. Moreover, the decision to treat a patient with abatacept was made before their enrollment in the study. By using a process of random selection from a comprehensive list of rheumatologists, the investigators in each country were geographically balanced and representative of rheumatologists who treat patients with biologics.

Enrolled patients provided informed written consent, were over 18 years of age, of either gender, with an established diagnosis of moderate-to-severe RA as defined by the American College of Rheumatology revised criteria 1987 [19]. Any patients already enrolled in an interventional RA clinical trial were excluded. The study protocol and patient enrollment were approved by ethics committees and regulatory agencies in accordance with each country’s requirements. The central ethics committee that first approved the study on 31 January 2008 was the Munich, Bavaria, Germany ethics committee. For each country, local ethics committee approvals were also obtained, as required by local regulations.

The ACTION study was conducted in accordance with the Declaration of Helsinki and was consistent with the International Conference on Harmonization Good Clinical Practice Guidelines [20] and Good Epidemiological Practice Guidelines [21].

Each patient was followed for up to 2 years or, if the patient discontinued abatacept treatment before the 2-year endpoint, for up to 6 months after abatacept discontinuation. Follow-up visits were approximately every 3 months. No formal assessment was performed to define reasons for prior treatment failure, other than those reported by the treating physician.

### Effectiveness assessments

Clinical characteristics and effectiveness are reported for patients with data available at baseline and Month 6, assessed no later than 8 days after the first abatacept infusion. Previous studies have demonstrated that abatacept may have an impact on efficacy measures as early as 7 days from the first infusion [22]. Patients who had their clinical assessment more than 8 days after their first abatacept infusion were not included in the effectiveness analysis. Disease activity was evaluated using the 28-item Disease Activity Score (DAS28), based either on erythrocyte sedimentation rate (ESR) or C-reactive protein (CRP) [23,24] according to physician’s choice, and Clinical Disease Activity Index (CDAI) [25]. Although investigators could report disease activity outcomes using the DAS28 and/or CDAI scores, in practice a majority of investigators reported only DAS28 scores; in addition, the CDAI score was calculated from core components collected for each patient. A sensitivity analysis was conducted on data from patients for whom both DAS28 and CDAI assessments were available and showed that the effectiveness outcomes at Month 6 in these patients were similar to those in the overall population. For DAS28, patients were classified as being in high (>5.1), moderate (>3.2 and ≤5.1), or low disease activity state (LDAS; ≤3.2), or remission (<2.6) [26]. LDAS was defined as a CDAI score ≤10, and remission was defined as a CDAI score ≤2.8. European League Against Rheumatism (EULAR) response was defined as good/moderate or no response and was based on DAS28 (ESR) or DAS28 (CRP) [27]. Physical function was assessed using the Health Assessment Questionnaire-Disability Index (HAQ-DI) [28]. HAQ response was defined as a mean change from baseline in HAQ score of ≥0.3 units [29]; a clinically meaningful change in physical function was defined as a mean change from baseline in HAQ score of ≥0.22 units [29-31].

### Safety assessments

Safety was evaluated in accordance with local regulations and registered with the drug manufacturer’s global pharmacovigilance department. Related treatment-emergent adverse events (AEs) were assessed by the treating physician and reported to the pharmacovigilance department. The relationship between the study drug and serious AE (SAE) was judged by the treating physician. A SAE was defined as an AE that was fatal or life-threatening, required or extended patient hospitalization (except pregnancy), resulted in persistent or significant disability or incapacity, induced a congenital anomaly or birth defect, or was considered an important medical event. All deaths were reported whether they were treatment-related or not. Safety was presented for the entire enrolled population, regardless of prior or concomitant treatment.

### Statistical analyses

The patient population was stratified by prior line of treatment into two subgroups: patients who were either biologic-naïve prior to initiating abatacept (‘first-line’), or patients who had previously received and failed at least one biologic agent (‘second-line’); this second group included patients initiating abatacept as a second- or further-line of treatment. Additional subgroup analyses of abatacept effectiveness and retention rates were performed for second-line patients stratified according to the number of prior anti-TNF agents failed (1 versus ≥2), the reason for discontinuing their previous biologic agent (primary/secondary inefficacy or safety and tolerability), or treatment pattern at abatacept initiation (monotherapy or in combination with conventional DMARDs). Baseline characteristics and demographics are presented using descriptive statistics for patients who received at least one infusion of abatacept and had data related to abatacept exposure. Retention on abatacept, defined as consecutive time on treatment, was analyzed using a Kaplan–Meier product limit estimator and is presented at Month 6 with 95% CIs. Patient discontinuation from abatacept treatment was recorded by the physician at any follow-up visit. In cases of abatacept discontinuation, exposure to abatacept was defined as the time between the date of the first abatacept infusion and the date of the last abatacept infusion, plus 30 days. Patients for whom data were not available at 6 months or who did not report abatacept discontinuation were censored at the date of the last available data. Effectiveness analyses were ‘as-observed’ for patients on treatment for whom data were available at each time point. These data were presented as proportions with 95% CIs, mean values, or changes with accompanying standard deviations (SDs) or 95% CIs.

## Results

### Patient disposition

**Figure 1:**
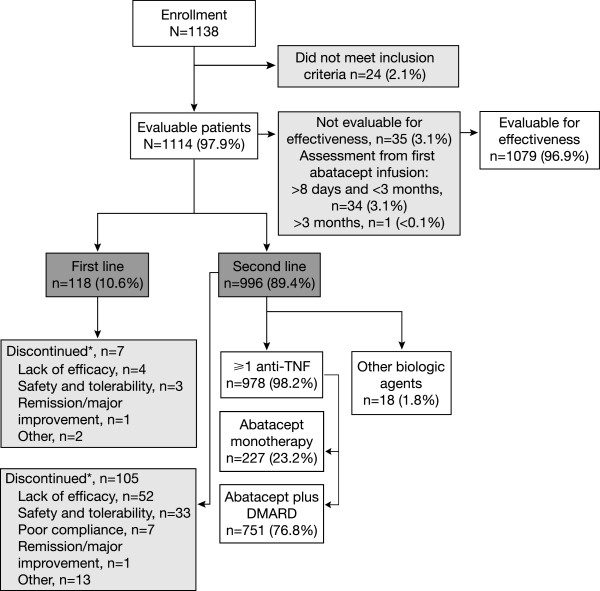
**Patient disposition.** *Patients could provide more than one reason for discontinuation. DMARD, disease-modifying anti-rheumatic drug; TNF, tumor necrosis factor.

Patient disposition is summarized in Figure 1. Of 1138 enrolled patients, 1114 (97.9%) were evaluable for the descriptive analysis and retention rate calculation. Patients were enrolled from nine countries (Austria, Belgium, Canada, Czech Republic, Denmark, Germany, Greece, Italy, The Netherlands) with the largest patient numbers enrolled in Germany (n = 399), Italy (n = 236), Canada (n = 229), and Greece (n = 149). A total of 96.9% (n = 1079/1114) of patients were evaluable for the effectiveness analysis; comprising patients who had a baseline clinical assessment on the same day/day before their first abatacept infusion (68.9% [n = 767]), ≥2 days before their first abatacept infusion (26.2% [n = 292]), ≤ 8 days after their first abatacept infusion (1.4% [n = 16]), and patients with the date of baseline clinical assessment missing (0.4% [n = 4]). Of the 35 (3.1%) patients who were not considered for the analysis of effectiveness outcomes, 34 (3.1%) had a baseline assessment between 8 days and 3 months following their first abatacept infusion and 1 (<0.1%) patient >3 months later. At the time of this analysis, approximately 86% of patients had outcomes at Month 6 and 14% had either been lost to follow-up at the data cut-off or their documentation had not been received. Patient and disease characteristics at abatacept initiation (baseline) were similar in patients with and without available data at Month 6. A total of 118 (10.6%) evaluable patients were stratified to the ‘first-line’ treatment group, and 996 (89.4%) were stratified to the ‘second-line’ treatment group.

A total of 112 patients in the overall population discontinued treatment within the first 6 months of the study; 7 patients were in the first-line group and 105 patients were in the second-line group. Reasons for discontinuation are shown in Figure 1.

### Characterization of the patient population

Baseline demographic characteristics of the evaluable patients were similar between patients in either line-of-treatment group (Table 1). Mean disease duration was shorter for patients in the first- versus second-line treatment group (6.9 versus 11.5 years (Table 1); there were more second-line treatment patients with disease duration longer than 6 years (68.8%) compared with the first-line treatment group (38.8%).

**Table 1:** Baseline demographics, disease and clinical characteristics by line-of-treatment category

**Demographic characteristics**	**N**	**First-line n = 118**	**Second-line n = 996**	**Overall n = 1114**
Age, mean (SD)	**1113**	59.1 (13.7)	56.2 (12.4)	56.5 (12.6)
Female, n (%)	**1114**	82 (69.5)	822 (82.5)	904 (81.1)
Weight, mean (SD)	**1102**	75.5 (15.9)	74.5 (17.0)	74.6 (16.9)
**Disease characteristics**				
Mean disease duration, years (SD)	**1079**	6.9 (7.7)	11.5 (8.9)	11.0 (8.9)
Disease duration, n (%)				
≤2 years		41 (35.3)	100 (10.4)	141 (13.1)
3–5 years		30 (25.9)	200 (20.8)	230 (21.3)
6–10 years		21 (18.1)	242 (25.1)	263 (24.4)
>10 years		24 (20.7)	421 (43.7)	445 (41.2)
**Previous treatments**				
Previously treated with biologic agents, n (%)	**1114**	0 (0.0)	996 (100)	996 (89.4)
At least one anti-TNF agent, n (%)		0 (0.0)	978 (98.2)	978 (87.8)
Anti-TNF only		0 (0.0)	790 (79.3)	790 (70.9)
Anti-TNF and another biologic		0 (0.0)	188 (18.9)	188 (16.9)
Other mechanisms of action only		0 (0.0)	18 (1.8)	18 (1.6)
Number of prior anti-TNF agents, mean (SD)		0 (0.0)	1.6 (0.7)	1.4 (0.8)
One, n (%)		0 (0.0)	480 (48.2)	480 (43.1)
Two, n (%)		0 (0.0)	405 (40.7)	405 (36.4)
Three, n (%)		0 (0.0)	93 (9.3)	93 (8.3)
**Clinical characteristics**	N*	**First-line N = 111**	**Second-line N = 968**	**Overall N = 1079**
Tender joint count (28), mean (SD)	**1052**	11.5 (7.3)	11.4 (7.3)	11.4 (7.3)
Swollen joint count (28), mean (SD)	**1069**	9.5 (5.8)	7.8 (5.8)	8.0 (5.9)
Patient global assessment, mean (SD) (VAS 100 mm)	**1002**	61.9 (22.1)	66.2 (20.1)	65.8 (20.3)
Physician global assessment, mean (SD) (VAS 100 mm)	**937**	61.9 (18.7)	61.8 (19.4)	61.8 (19.3)
Patient global assessment of pain, mean (SD) (VAS 100 mm)	**990**	59.9 (24.5)	65.9 (20.7)	65.3 (21.1)
Patients with erosions, n (%)	**926**	58 (58.0)	590 (71.4)	648 (70.0)
DAS28 (ESR), mean (SD)	**748**	5.5 (1.3)	5.6 (1.2)	5.6 (1.2)
DAS28 (CRP), mean (SD)	**216**	4.8 (1.1)	5.2 (1.3)	5.2 (1.3)
CDAI, mean (SD)	**919**	33.4 (13.1)	31.5 (13.0)	31.7 (13.0)
SDAI, mean (SD)	**824**	35.4 (13.8)	33.9 (13.8)	34.0 (13.8)
HAQ-DI, mean (SD)	**988**	1.42 (0.59)	1.56 (0.67)	1.55 (0.67)
CRP mg/L, mean (SD)	**943**	19.6 (32.5)	24.4 (40.6)	23.9 (39.9)
ESR mm/hour, mean (SD)	**988**	32.5 (23.6)	35.5 (24.5)	35.2 (24.4)
Rheumatoid factor positive, n (%)	**886**	64 (68.1)	549 (69.3)	613 (69.2)
Anti-CCP positive, n (%)	**598**	36 (59.0)	354 (65.9)	390 (65.2)

*All patients with relevant baseline data assessed no later than 8 days after the first infusion of abatacept.

CCP, anti-cyclic citrullinated protein; CDAI, Clinical Disease Activity Index; CRP, C-reactive protein; DAS28, Disease Activity Score 28; ESR, erythrocyte sedimentation rate; HAQ-DI, Health Assessment Questionnaire-Disability Index; SD, standard deviation; SDAI, Simplified Disease Activity Index; TNF, tumor necrosis factor; VAS, visual analog scale.

The mean number of non-biologic DMARDs received prior to enrolling in the study was 2.2 in the first-line group received and 2.9 in the second-line group. Prior treatment with corticosteroids or MTX was reported for high and similar proportions of first- and second-line patients (Table 1). Of the patients previously treated with biologics (n = 996), 79.3% (n = 790) had previously failed anti-TNF treatment only, and 18.9% (n = 188) had treatment failures with both an anti-TNF and a non-anti-TNF biologic. Of the 823 (84.1%) patients who received anti-TNF therapy before enrolling in the study, 41.2% (n = 339) received adalimumab, 40.2% (n = 331) etanercept, 17.1% (n = 141) infliximab, 0.9% (n = 7) certolizumab, and 0.6% (n = 5) golimumab. Of patients receiving treatment with non-anti-TNF biologics prior to study enrollment, 9.7% (n = 95) were treated with rituximab and 4.6% (n = 45) with tocilizumab.

The mean number of biologic treatment failures for second-line patients prior to initiating abatacept was 1.8. Among the 974 patients for whom reasons for discontinuation of the prior biologic before study enrollment were available, the reasons were: primary inefficacy (26.6%, n = 259), loss of efficacy (secondary inefficacy) (46.5%, n = 453), safety and tolerability (22.0%, n = 214), other unspecified reasons (5.6%, n = 55), and achieving remission or experiencing a major improvement (0.5%, n = 5). Patients could report more than one reason for discontinuation of prior treatment before study enrollment.

In the first-line treatment group (n = 118), 7.6% (n = 9) of patients initiated abatacept as monotherapy and 92.4% (n = 109) received it in combination with another DMARD, which was MTX in 42.2% of patients (n = 46). Abatacept was initiated in combination with corticosteroids in 64.4% (n = 76) of first-line patients. Of the 996 patients who had previously failed at least one biologic agent, 22.8% (n = 227) received abatacept as monotherapy and 77.2% (n = 769) received abatacept in combination with another DMARD, of whom 61.0% (n = 469) received abatacept in combination with MTX. Abatacept was initiated in combination with corticosteroids in 74.9% (n = 746) of patients in the second-line treatment group.

Patient clinical characteristics by line-of-treatment group are summarized in Table 1. The majority of patients were at high risk of disease progression: 58.0% in the first-line group and 71.4% in the second-line group had erosions, 59.0% of first-line and 65.9% of second-line patients were anti-cyclic citrullinated protein positive, and 68.1% in the first-line and 69.3% in the second-line group were rheumatoid factor (RF) positive. Overall, patients in both treatment groups had high levels of disease severity at baseline according to DAS28 (ESR), CDAI, and HAQ-DI scores (Table 1).

Similar proportions of patients from both groups presented with at least one comorbidity at enrollment; most commonly metabolic disorders (26.8%, n = 298), including lipid metabolism and deposit disorders (not elsewhere classified [15.9%, n = 177]), and diabetes (12.1%, n = 135); endocrine disorders (14.2%, n = 158), including hypothyroidism (10.1%, n = 112); respiratory disease (10.1%, n = 113); and cardiac disorders (6.4%, n = 71). Infections and infestations were reported by 5.9% (n = 66) of patients, including 1.4% (n = 16) of patients with tuberculosis. Other comorbidities at baseline included hepatobiliary disorders (2.3%, n = 26), renal disorders (2.4%, n = 27), and neoplasms (benign, malignant, and unspecified; 2.6%, n = 29).

### Retention rate

**Figure 2:**
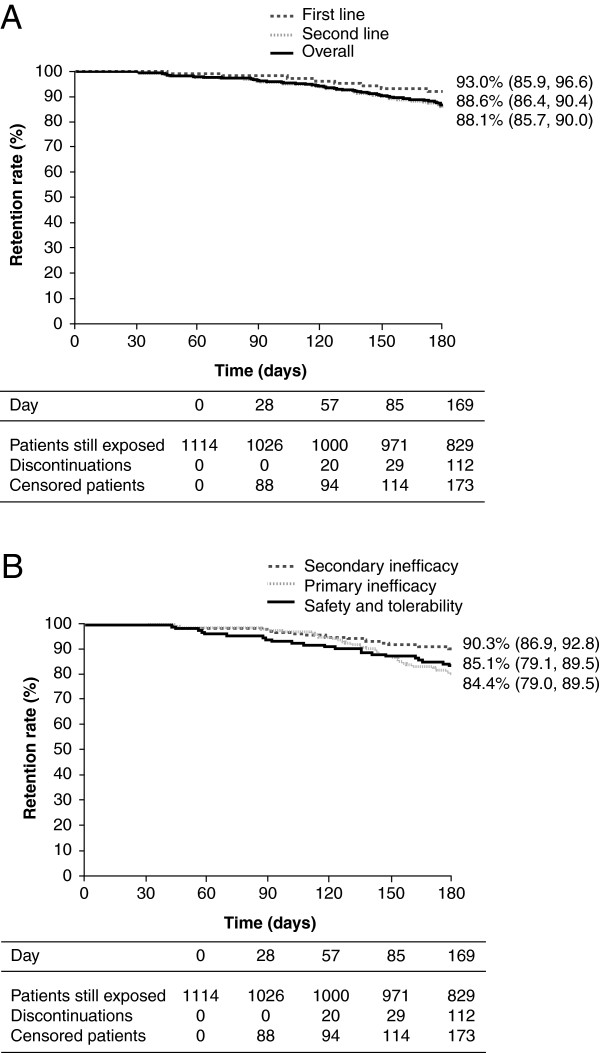
**Kaplan–Meier estimated patient retention rates at month 6.** The estimate is given for each patient group with 95% CIs. **(A)** Kaplan–Meier curves for the overall patient population, the first-line group and the second-line group. **(B)** Kaplan–Meier curves based on reasons for discontinuation of prior biologic therapy are shown for patients who discontinued due to primary inefficacy, secondary inefficacy, or safety and tolerability reasons. CI, confidence interval.

Retention rates in abatacept-treated patients are shown in Figure 2. The Kaplan–Meier estimated retention rate at endpoint (Day 169/Month 6) for all evaluable patients (n = 1114) treated with abatacept was 88.6% (95% CI: 86.4, 90.4). For those in the first-line group, the retention rate was 93.0% (95% CI: 85.9, 96.6), whereas for patients in the second-line group it was 88.1% (95% CI: 85.7, 90.0) (Figure 2A).

For patients in the second-line group, the Kaplan–Meier estimated retention rate at endpoint (Day 169/Month 6) for patients initiating abatacept treatment after >1 prior failed anti-TNF treatment was 89.2% (95% CI: 85.8, 91.8) and for those who had failed ≥2 anti-TNF therapies it was 86.7% (95% CI: 83.1, 89.5). The Kaplan–Meier estimated retention rates based on reasons for discontinuing prior biologic therapy before initiating abatacept were 84.4% (95% CI: 79.0, 88.6) for patients who discontinued due to primary inefficacy, 90.3% (95% CI: 86.9, 92.8) for those who discontinued due to secondary inefficacy, and 85.1% (95% CI: 79.1, 89.5) for those with safety and tolerability issues with anti-TNF agents (Figure 2B). The estimated retention rate was 87.7% (95% CI: 82.4, 91.5) for patients in the second-line group who had received abatacept monotherapy and 88.1% (95% CI: 85.4, 90.4) for patients who had received abatacept in combination with a DMARD at initiation.

### Effectiveness over 6 months

Changes in disease state were assessed using the DAS28 (ESR), DAS28 (CRP), and CDAI scores for patients in the overall population with data evaluable for effectiveness at baseline and Month 6. Mean (SD) baseline DAS28 (ESR), DAS28 (CRP), and CDAI scores were 5.5 (1.2), 5.2 (1.3), and 31.7 (13.2), respectively, and mean (95% CI) changes from baseline at Month 6 were −1.5 (−1.6, –1.3), –1.5 (−1.7, –1.2), and −15.2 (−16.3, –14.1), respectively (Table 2). Patients receiving abatacept earlier in the course of treatment (ie, first-line) achieved numerically greater mean changes from baseline in DAS28 (ESR), DAS28 (CRP), and CDAI compared with second-line abatacept, although 95% CI overlapped (Table 2). Among second-line patients, mean changes from baseline in DAS28 (ESR), DAS28 (CRP), and CDAI were numerically greater among those who failed one prior anti-TNF and those who failed ≥2, but with overlapping 95% CI (Table 2).

**Table 2:** Analysis of abatacept effectiveness overall, and stratified by line of therapy, and by number of previous failed anti-TNFs

**Measure**	**Baseline**		**Change from baseline to month 6**	
	**N**	**Mean (SD)**		**Mean (95% CI)**
		**Overall**		
**DAS28 (ESR)**	539	5.5 (1.2)	473	−1.5 (−1.6, –1.3)
**DAS28 (CRP)**	151	5.2 (1.3)	113	−1.5 (−1.7, –1.2)
**CDAI**	647	31.7 (13.2)	605	−15.2 (−16.3, –14.1)
		**First-line**		
**DAS28 (ESR)**	33	5.2 (1.2)	29	−1.7 (−2.3, –1.1)
**DAS28 (CRP)**	5	4.5 (1.3)	4	−2.0 (−3.2, –0.8)
**CDAI**	48	31.9 (11.9)	41	−18.3 (−22.0, –14.6)
		**Second-line**		
**DAS28 (ESR)**	506	5.6 (1.2)	444	−1.5 (−1.6, –1.3)
**DAS28 (CRP)**	146	5.3 (1.3)	109	−1.4 (−1.7, –1.2)
**CDAI**	599	31.7 (13.2)	564	−15.0 (−16.1, –13.9)
		**One previous anti-TNF**		
**DAS28 (ESR)**	247	5.5 (1.2)	221	−1.6 (−1.8, –1.4)
**DAS28 (CRP)**	64	5.2 (1.3)	46	−1.7 (−2.2, –1.2)
**CDAI**	291	30.6 (12.7)	278	−15.0 (−16.5, –13.5)
		**≥2 previous anti-TNFs**		
**DAS28 (ESR)**	251	5.6 (1.3)	216	−1.3 (−1.5, –1.1)
**DAS28 (CRP)**	78	5.3 (1.4)	59	−1.2 (−1.6, –0.8)
**CDAI**	295	32.5 (13.6)	275	−14.7 (−16.4, –12.9)

CDAI, Clinical Disease Activity Index; CI, confidence interval; CRP, C-reactive protein; DAS28, Disease Activity Score 28; ESR, erythrocyte sedimentation rate; LDAS, low disease activity state; SD, standard deviation; TNF, tumor necrosis factor.

**Figure 3:**
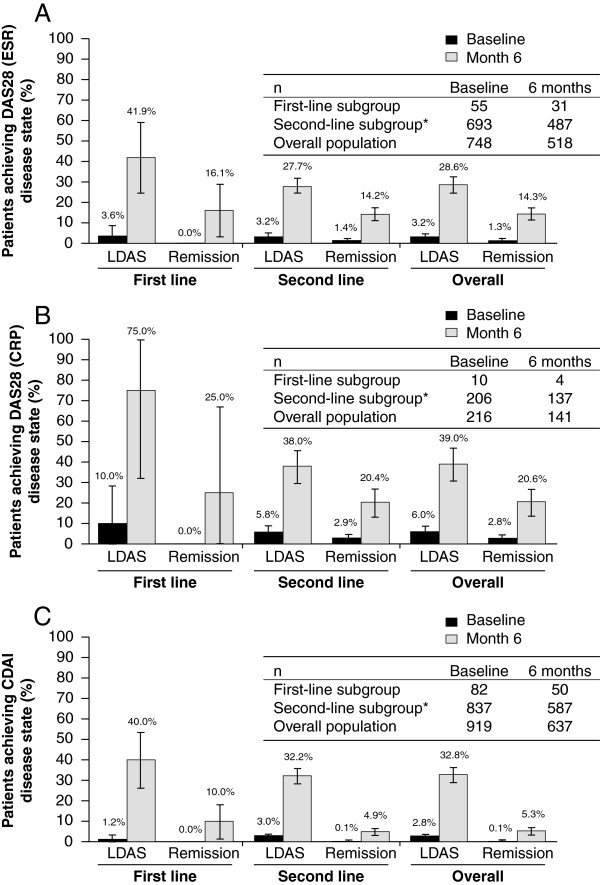
**Proportion of patients with LDAS or remission at baseline and at month 6. (A)** Assessed by the DAS28 (ESR), **(B)** by the DAS28 (CRP), and **(C)** by the CDAI. LDAS was defined as a DAS28 score ≤3.2 or a CDAI score ≤10. Remission was defined as a DAS28 score <2.6 or a CDAI score ≤2.8. Error bars represent 95% CI. *Includes patients receiving abatacept as a second- or further-line of treatment. CDAI, Clinical Disease Activity Index; CI, confidence interval; CRP, C-reactive protein; DAS28, 28-item Disease Activity Score; ESR, erythrocyte sedimentation rate; LDAS, low disease activity state.

The proportions of patients achieving LDAS or remission are shown in Figure 3A–C. The proportions achieving LDAS or remission were higher by the DAS–CRP criteria. By all criteria, a numerically higher proportion of first-line patients achieved both LDAS and remission compared with second-line patients. Among second-line patients, a numerically higher proportion of patients who failed one prior anti-TNF had achieved LDAS and remission, for all three composite scales, compared with patients who had failed ≥2 prior anti-TNFs although 95% CIs overlapped (Table 3).

**Table 3:** Proportion of patients with LDAS* or remission at baseline and at Month 6 for second line abatacept stratified by 1 or ≥2 **prior anti-TNF agent**

**Measure**	**Remission at month 6**		LDAS* at month 6	
	**N**	**Percent (95% CI)**	**N**	**Percent (95% CI)**
		**One previous anti-TNF**		
**DAS28 (ESR)**	245	15.9 (11.3, 20.5)	245	29.8 (24.1, 35.5)
**DAS28 (CRP)**	57	22.8 (11.9, 33.7)	57	42.1 (29.3, 54.9)
**CDAI**	289	5.2 (2.6, 7.7)	289	35.6 (30.1, 41.2)
		**≥2 previous anti-TNFs**		
**DAS28 (ESR)**	234	12.8 (8.5, 17.1)	234	25.6 (20.0, 31.2)
**DAS28 (CRP)**	75	17.3 (8.8, 25.9)	75	33.3 (22.7, 44.0)
**CDAI**	286	4.9 (2.4, 7.4)	286	28.3 (23.1, 33.5)

*LDAS includes patients in remission (DAS remission: <2.6; CDAI remission: ≤2.8).

CDAI, Clinical Disease Activity Index; DAS, Disease Activity Score; LDAS, Low Disease Activity Score (≤3.2 for DAS and ≤10 for CDAI).

**Figure 4:**
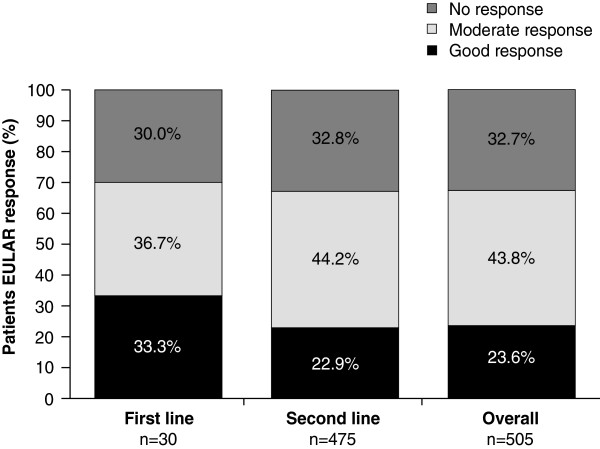
**Proportion of patients achieving a response at month 6 as defined by the EULAR.** EULAR, European League Against Rheumatism.

The proportion of patients achieving a EULAR response is shown in Figure 4. More than 67% of patients achieved a good or moderate EULAR response, as defined by DAS28 (ESR) or DAS28 (CRP) independently of whether abatacept was initiated as first- or second-line therapy (Figure 4). A good or moderate EULAR response was achieved by similar proportions of patients regardless of whether they had previously failed 1 or ≥2 anti-TNFs (69.2 and 64.5%, respectively).

Effectiveness was also stratified by reason for discontinuation of patients with prior biologic therapy. Mean change from baseline in DAS28 (ESR), DAS28 (CRP), and CDAI was similar in patients who discontinued their prior biologic due to primary inefficacy, secondary inefficacy, or safety and tolerability issues (Table 4). Slightly different results were observed when considering LDAS and remission (Table 4).

**Table 4:** Subgroup analysis of second-line abatacept effectiveness by reasons for treatment failure

**Measure**	**Baseline**		**Change from baseline to month 6**		**Remission at month 6**		**LDAS at month 6**	
	**N**	**Mean (SD)**	**N**	**Mean (95% CI)**	**N**	**Percent (95% CI)**	**N**	**Percent (95% CI)**
			**Primary inefficacy**					
**DAS28 (ESR)**	129	5.6 (1.3)	113	−1.5 (−1.7, –1.2)	119	17.6 (10.8, 24.5)	119	29.4 (21.2, 37.6)
**DAS28 (CRP)**	35	5.2 (1.3)	26	−1.3 (−2.0, –0.7)	32	12.5 (1.0, 24.0)	32	34.4 (17.9, 50.8)
**CDAI**	140	32.1 (13.1)	129	−13.9 (−16.3, –11.5)	136	2.9 (0.1, 5.8)	136	27.9 (20.4, 35.5)
			**Secondary inefficacy**					
**DAS28 (ESR)**	249	5.6 (1.1)	219	−1.4 (−1.6, –1.2)	240	10.4 (6.6, 14.3)	240	25.0 (19.5, 30.5)
**DAS28 (CRP)**	74	5.5 (1.3)	55	−1.6 (−2.0, –1.3)	68	20.6 (11.0, 30.2)	68	38.2 (26.7, 49.8)
**CDAI**	295	32.5 (13.0)	279	−15.4 (−17.1, –13.8)	288	4.2 (1.9, 6.5)	288	29.5 (24.2, 34.8)
			**Safety and tolerability**					
**DAS28 (ESR)**	91	5.5 (1.3)	84	−1.5 (−1.8, –1.2)	98	18.4 (10.7, 26.0)	98	29.6 (20.6, 38.6)
**DAS28 (CRP)**	27	4.8 (1.4)	22	−1.5 (−2.2, –0.9)	26	30.8 (13.0, 48.5)	26	42.3 (23.3, 61.3)
**CDAI**	121	30.4 (13.4)	115	−15.2 (−17.6, –12.8)	119	8.4 (3.4, 13.4)	119	38.7 (29.9, 47.4)

CDAI, Clinical Disease Activity Index; CRP, C-reactive protein; DAS, Disease Activity Score; ESR, erythrocyte sedimentation rate.

A good or moderate EULAR response was achieved by similar proportions of patients regardless of the reasons for which the last biologic therapy was discontinued (67.2, 66.2, and 67.3% for those patients who discontinued due to primary inefficacy, secondary inefficacy, or intolerance, respectively). When comparing abatacept as monotherapy versus in combination with a DMARD in patients treated with abatacept as second-line or higher, 63.1% (n = 65/103) versus 68.3% (n = 254/372) of patients achieved a good or moderate EULAR response, 27.5% (n = 38/138) versus 33.6% (n = 151/449) achieved CDAI LDAS, and 5.1% (n = 7/138) versus 4.9% (n = 22/449) achieved CDAI remission.

Among patients for whom data were available at Month 6, the mean (SD) baseline HAQ-DI was 1.5 (0.6), and the mean change in HAQ-DI score from baseline to Month 6 was −0.30 (95% CI: –0.35, –0.26). After 6 months of abatacept treatment, 44.7% achieved a HAQ response (≥0.3 unit change from baseline), and 55.0% of patients achieved a clinically meaningful change (≥0.22 unit change from baseline). Patients receiving abatacept earlier in the course of treatment (ie, first-line) achieved numerically greater mean changes from baseline in HAQ-DI compared with second-line abatacept (−0.44, [95% CI: –0.58, –0.29] versus −0.29 [95% CI: –0.34, –0.24], respectively), although 95% CIs overlapped. A greater proportion of first-line patients achieved a HAQ-DI response compared with second-line patients (60.3% [95% CI: 47.8, 72.9] versus 43.1% [95% CI: 39.0, 47.2], respectively). Among second-line patients, the mean change from baseline in HAQ-DI (−0.35 [95% CI: –0.42, –0.28] versus −0.23 [95% CI: –0.29, –0.17]) and the proportion of patients achieving a HAQ-DI response (48.5 [95% CI: 42.6, 54.5] versus 37.1% [95% CI: 31.4, 42.8]) were greater among those who failed one prior anti-TNF compared to those who failed ≥2.

### Concomitant medication

Overall, 822/1114 (73.8%) and 555/770 (72.1%) of patients were receiving concomitant corticosteroids at abatacept initiation and at 6 months, respectively. The median dose decreased from 7.5 mg/day (n = 724) to 5 mg/day (n = 494) over 6 months. Among those who were on concomitant corticosteroids at abatacept initiation and for whom 6-month data were available, 39/555 (7.0%) of patients discontinued all corticosteroids from initiation to 6 months, and 141/462 (30.5%) patients had a dose decrease from 10 mg/day (median dose at initiation) to 5 mg/day (median dose at 6 months). Among patients who were not on concomitant corticosteroids at abatacept initiation and for whom 6-month data were available, 39/215 (18.1%) of patients had concomitant corticosteroids introduced to their treatment regimen between abatacept initiation and Month 6; the median dose at Month 6 was 7.5 mg/day. From initiation to Month 6, 30/770 (3.9%) patients discontinued all concomitant DMARDs, whereas 20/770 (2.6%) patients had concomitant DMARDs introduced during the first 6 months after abatacept initiation.

### Safety

Safety was reported for all 1138 enrolled patients, and no new or unexpected AEs were reported. SAEs were reported in 4.7% (n = 54/1138) of patients and discontinuations due to SAEs occurred in 1.8% (n = 20/1138) of patients. Nine deaths were reported throughout the study. Causes of death (n = 1, each) were: aspiration pneumonia secondary to withdrawal from benzodiazepines, asthma and stroke, seizure, heart attack, urosepsis, suicide, *Pneumocystis jiroveci* pulmonary infection, sepsis, and unknown.

Serious infections were reported in 1.7% (n = 19) of patients. No cases of active tuberculosis were reported and one case of opportunistic infection (*Pneumocystis jiroveci*) was reported but not confirmed by culture. Investigators considered these infections to be unrelated to treatment. Nine patients presented with malignancies during the study that were not considered related to treatment. Five patients had serious cardiac disorders and three had vascular disorders (stroke, transient ischemic event, and deep-vein thrombosis). Diverticular perforation resulting in sepsis was reported in one patient, for which surgery was performed. One severe acute systemic infusion reaction as the result of an allergic reaction was reported 25 minutes after beginning an abatacept infusion. Pulmonary disorders were reported in seven patients during the study, including one patient with an event of bronchitis, who had known pre-existing risk factors (tobacco use and grade II chronic obstructive pulmonary disease).

## Discussion

ACTION was the first international, non-interventional, multicenter, prospective cohort study to evaluate patient retention and effectiveness of abatacept treatment in patients with moderate-to-severe RA. The current interim analysis evaluated a 6-month dataset from this ongoing 2-year study. This 6-month interim analysis may be particularly pertinent to clinicians because, according to the treat-to-target approach, the decision to switch a biologic therapy is usually made 3–6 months after initiating treatment. Here, we demonstrate high patient retention on abatacept, efficacy benefits with regards to disease activity and physical function, and a safety profile consistent with observations from both RCTs and local national registries. Benefits were observed in biologic-naïve and anti-TNF-refractory patients, regardless of the number of previously failed anti-TNF agents, or whether failure was due to primary or secondary inefficacy, or safety and tolerability reasons. In the current study, approximately 70% of enrolled patients were RF positive, which is consistent with the proportion of RF-positive patients enrolled in abatacept RCTs (ATTAIN study, 73.3%; ARRIVE, 61.3%) [13,32] and in real-life abatacept studies (ORA, 72.5%) [33].

It has been reported that treatment response rates are often lower in routine clinical practice compared with RCT evidence [7], as a result of the patient populations in observational studies not being subject to the strict inclusion and exclusion criteria of RCTs. However, the heterogeneity of patient populations and disease characteristics in observational studies provide a real-world perspective of routine clinical practice. The efficacy, safety, and tolerability of abatacept for the treatment of moderate-to-severe RA have been demonstrated in RCTs [10-14], in local national registries [15,16], and in a small, single-site observational study [18]. Therefore, the objective of the ACTION study was to translate the validity of RCT results into a real-life setting. Given the objective of the study, a single-arm design was considered appropriate to describe a cohort of patients treated with abatacept and assess their drug utilization in accordance with the European Medicines Agency and Health Technology Assessment Programmes’ recommendations.

Retention rates reported in the current trial were high – >80.0% for second-line and 93.0% for first-line patients – compared with evidence from other real-world observational studies. Evidence from the Swedish national registry ARTIS showed that, 1 year after initiating abatacept treatment, retention rates were 80% for biologic-naïve patients and 64% for patients previously treated with 1–2 biologics [17]. Similarly, 6-month retention rates with abatacept treatment were 72.0% in the Danish DANBIO registry [16] and 80.0% in a US observational study [18]. Although retention rates for RCTs are expected to be higher than for real-world studies [7], the retention rates in the current study were consistent with an 82–90% retention rate reported from two abatacept RCTs, the ATTAIN and ARRIVE studies [13,32]. Of note was the high retention rate for biologic-naïve patients in the current study (93.0%), which is consistent with evidence from abatacept RCTs showing that patient retention is higher when abatacept is initiated earlier in the treatment regimen [11,14].

The efficacy of abatacept in the current report was assessed using multiple disease activity measures (DAS28, CDAI, and EULAR response); each of the clinical indices showed the same trend for improved effectiveness with abatacept, including the CDAI. Changes in disease activity in the current study were consistent with those reported in the DANBIO national registry [16] and the French ORA registry [33]. A good-to-moderate treatment response, as defined by EULAR, was achieved by more than 67% of both first- and second-line patients in the current analysis; this was consistent with 6-month evidence from the French ORA national registry [33] and the Danish DANBIO registry [16]. In addition, response and remission rates with abatacept in the ACTION study were similar to those reported in the two previously mentioned abatacept RCTs in patients with a prior inadequate response to anti-TNF agents, the ATTAIN [13] and ARRIVE studies [32]. Subgroup analysis from ACTION – according to the number of prior anti-TNFs failed or according to the reason for discontinuation of the last biologic prior to abatacept – highlight that abatacept has favorable safety and tolerability in a real-world setting, regardless of the number of prior anti-TNFs failed or the reason for failure. These data support previously reported favorable outcomes from the ARRIVE [32] trial in patients with similar characteristics. Furthermore, the subgroup analyses in ACTION showed consistent numerically superior outcomes for patients treated with abatacept earlier in their disease course. As the study was not powered for subgroups analysis, definitive conclusions cannot be drawn.

Overall, no new safety signals were identified in the ACTION patient population compared with the safety profile previously reported for abatacept from real-world studies [16,18]. Of note is the absence of any cases of active tuberculosis and one report of opportunistic infection. It is important to note that a large number of patients enrolled in the ACTION study had cardiovascular and pulmonary comorbidities, as well as chronic infections, at baseline, reflecting the type of patient profile often found in routine clinical practice compared with RCTs.

Although the results of some observational studies indicate that, after the failure of 1 or ≥2 anti-TNF agents, the choice of a biological agent with a different mechanism of action may lead to better clinical outcomes, there are a number of limitations associated with such analyses [34]. To our knowledge, there is little evidence from real-life settings that directly compares abatacept with another biologic agent or biologic agents with each other. When interpreting the results of the current study, there are a number of potential limitations to be considered including lack of an active comparator and/or selection bias based on factors such as disease severity or AEs. In addition, failure of multiple biologics prior to abatacept treatment may have influenced physicians to wait longer before deciding that a treatment was ineffective, potentially affecting the retention rate by Month 6. This interim analysis was also vulnerable to missing data as none of the study assessments were mandatory; thus, most missing clinical outcome data may have been attributed to assessments not performed routinely at all locations. Consequently, of the 24.7% of patients with missing numerical DAS28 scores, 15.9% were reported by the investigators as score ‘not calculated’. Here, we report data ‘as observed’ with no imputation for missing values, which is consistent with other non-interventional studies.

## Conclusions

This large, observational, real-world study demonstrated high patient retention rates with abatacept treatment, regardless of line of treatment (first or second), the number of previously failed anti-TNF agents, or the reason for treatment failure. In addition, the data suggest that patients treated earlier in their disease course with abatacept have better outcomes than patients treated after failure of one or more anti-TNF agents. Rates of retention, LDAS, remission, HAQ-DI response, and safety outcomes were consistent with data from both abatacept RCTs and local national registries. Furthermore, increased proportions of patients achieved remission or LDAS after 6 months of abatacept treatment following the failure of <2 anti-TNF agents, compared with those who had failed ≥2 anti-TNF agents. The findings presented here underline that abatacept, when used alone or in combination with DMARDs, provides a well-tolerated and effective treatment option for patients with RA, including those for whom previous anti-TNF treatment has failed. These data further support the use of abatacept monotherapy in clinical practice, as reflected by observations from RA registries [6,33]. Future analyses of the ACTION study will evaluate the long-term effectiveness, retention rates, and safety of abatacept in the real-world setting.

## Abbreviations

ACTION: AbataCepT In rOutiNe clinical practice; AE: Adverse event; CDAI: Clinical disease activity index; CI: Confidence interval; CRP: C-reactive protein; DAS28: 28-item disease activity score; DMARD: Disease-modifying anti-rheumatic drug; ESR: Erythrocyte sedimentation rate; EULAR: European league against rheumatism; HAQ-DI: Health assessment questionnaire-disability index; LDAS: Low disease activity state; MTX: Methotrexate; RA: Rheumatoid arthritis; RCT: Randomized clinical trials; RF: Rheumatoid factor; SAE: Serious adverse event; SD: Standard deviation; TNF: Tumor necrosis factor.

## Competing interests

H.G. Nüßlein has received consulting fees and speaker honoraria from Bristol-Myers Squibb, Abbott, Chugai, UCB, Essex, Wyeth, Pfizer, MSD, Novartis, and Roche. R. Alten has received research grants from Bristol-Myers Squibb, Merck Pharma GmbH, Novartis, UCB, Roche, and Pfizer; speaker honoraria from Abbott Laboratories, Bristol-Myers Squibb, Horizon Pharma, Novartis, and Roche; and consulting fees from Abbott Laboratories, Horizon Pharma, Novartis, and Roche. M. Galeazzi has nothing to disclose. H.-M. Lorenz has received honoraria from Bristol-Myers Squibb for presentations and participation in advisory boards, and provides advice on planning of the ACTION study. D. Boumpas has nothing to disclose. M.T. Nurmohamed has received consultancy fees from Abbott, Roche, Pfizer, MSD, UCB, SOBI, and Bristol-Myers Squibb, and has received payment for lectures from Abbott, Roche, Bristol-Myers Squibb, and Pfizer. W.G. Bensen has attended advisory boards, presented data, and performed research for Bristol-Myers Squibb, Amgen, Abbott, UCB, Merck, Pfizer, Novartis, AstraZeneca, Roche, Janssen, Warner Chilcott, and Sanofi-Aventis. G.R. Burmester has received consulting fees from Abbott Immunology Pharmaceuticals, BMS, Roche, Merck, and Pfizer; research grants from Abbott Laboratories, Abbott Immunology Pharmaceuticals, Bristol-Myers Squibb, and Roche; and speaker honoraria from Abbott Immunology Pharmaceuticals, Bristol-Myers Squibb, and Roche. H.-H. Peter has nothing to disclose. F. Rainer has nothing to disclose. K. Pavelka has been a speaker for Pfizer, Amgen, MSD, Bristol-Myers Squibb, and Abbott. M. Chartier is a consultant for Bristol-Myers Squibb. C. Poncet is a consultant for Bristol-Myers Squibb. C. Rauch is an employee of Bristol-Myers Squibb. M. Le Bars is an employee of Bristol-Myers Squibb and holds stock options.

## Authors’ contributions

HGN made a substantial contribution to the conception and design of the study, to the acquisition of data, and to the analysis and interpretation of data. RA made a substantial contribution to the conception and design of the study, and to the analysis and interpretation of data. MG made a substantial contribution to the acquisition of data, and to the analysis and interpretation of data. HML made a substantial contribution to the conception and design of the study, to the acquisition of data, and to the analysis and interpretation of data. DB made a substantial contribution to the conception and design of the study, to the acquisition of data, and to the analysis and interpretation of data. MTN made a substantial contribution to the acquisition of data. WGB made a substantial contribution to the acquisition of data. GRB made a substantial contribution to the acquisition of data, and to the analysis and interpretation of data. HHP made a substantial contribution to the acquisition of data, and to the analysis and interpretation of data. FR made a substantial contribution to the acquisition of data. KP made a substantial contribution to the acquisition of data. MC made a substantial contribution to the conception and design of the study, to the acquisition of data, and to the analysis and interpretation of data. CP made a substantial contribution to the conception and design of the study, and to the analysis and interpretation of data. CR made a substantial contribution to the conception and design of the study, to the acquisition of data, and to the analysis and interpretation of data. MLB made a substantial contribution to the conception and design of the study, to the acquisition of data, and to the analysis and interpretation of data. All authors have read and approved the final manuscript.

## Pre-publication history

The pre-publication history for this paper can be accessed here:

http://www.biomedcentral.com/1471-2474/15/14/prepub
